# Control of Orthodontic Tooth Movement by Nitric Oxide Releasing Nanoparticles in Sprague-Dawley Rats

**DOI:** 10.3389/fmats.2022.811251

**Published:** 2022-04-14

**Authors:** Derrick Crawford, Tommy C. Lau, Megan C. Frost, Nan E. Hatch

**Affiliations:** 1 Department of Orthodontics and Pediatric Dentistry, School of Dentistry, University of Michigan, Ann Arbor, MI, United States,; 2 Department of Kinesiology and Integrative Physiology, Michigan Technological University, Houghton, MI, United States

**Keywords:** orthodontic, nitric oxide, biomaterial, nanoparticle, tooth movement, controlled release, rodent model

## Abstract

Orthodontic treatment commonly requires the need to prevent movement of some teeth while maximizing movement of other teeth. This study aimed to investigate the influence of locally injected nitric oxide (NO) releasing nanoparticles on orthodontic tooth movement in rats.

**Materials and Methods:**

Experimental tooth movement was achieved with nickel-titanium alloy springs ligated between the maxillary first molar and ipsilateral incisor. 2.2 mg/kg of silica nanoparticles containing S-nitrosothiol groups were injected into the mucosa just mesial to 1^st^ molar teeth immediately prior to orthodontic appliance activation. NO release from nanoparticles was measured *in vitro* by chemiluminescence. Tooth movement was measured using polyvinyl siloxane impressions. Bones were analyzed by microcomputed tomography. Local tissue was assessed by histomorphometry.

**Results:**

Nanoparticles released a burst of NO within the first hours at approximately 10 ppb/mg particles that diminished by 10 × to approximately 1 ppb/mg particles over the next 1–4 days, and then diminished again by tenfold from day 4 to day 7, at which point it was no longer measurable. Molar but not incisor tooth movement was inhibited over 50% by injection of the NO releasing nanoparticles. Inhibition of molar tooth movement occurred only during active NO release from nanoparticles, which lasted for approximately 1 week. Molar tooth movement returned to control levels of tooth movement after end of NO release. Alveolar and long bones were not impacted by injection of the NO releasing nanoparticles, and serum cyclic guanosine monophosphate (cGMP) levels were not increased in animals that received the NO releasing nanoparticles. Root resorption was decreased and periodontal blood vessel numbers were increased in animals with appliances that were injected with the NO releasing nanoparticles as compared to animals with appliances that did not receive injections with the nanoparticles.

**Conclusion:**

Nitric oxide (NO) release from S-nitrosothiol containing nanoparticles inhibits movement of teeth adjacent to the site of nanoparticle injection for 1 week. Additional studies are needed to establish biologic mechanisms, optimize efficacy and increase longevity of this orthodontic anchorage effect.

## INTRODUCTION

**FIGURE 1 |:**
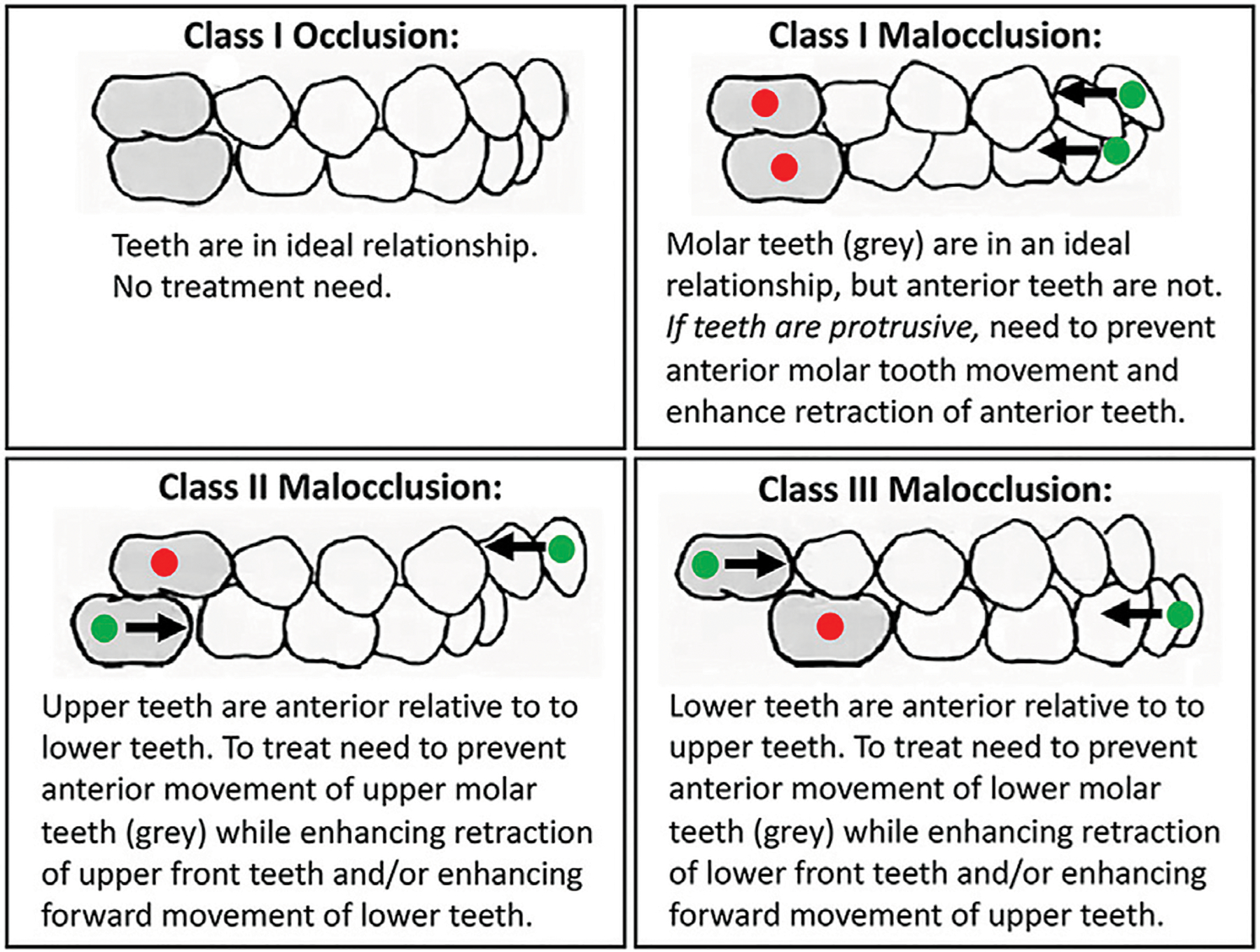
Many malocclusions require differential orthodontic tooth movement to achieve ideal functional and esthetic outcomes. Teeth that need inhibition of movement for successful treatment are marked with a red dot. Teeth that need enhancement of movement for successful treatment are marked with a green dot. Arrows mark direction of desired tooth movement.

Orthodontic treatment is common because untreated malocclusion negatively impacts oral function (eating, speech) and causes physical, psychological and social disability (Sun et al., 2017). Approximately 15% of the U.S. population has a severe dental malocclusion that negatively impacts function and social acceptability (Proffit et al., 1998). This translates to a prevalence of over 48 million people in the U.S. with significant orthodontic treatment need. Treatment of such severe cases requires precise control of individual teeth, including the ability to prevent movement of some teeth while maximizing movement of other teeth (Figure 1). In orthodontics, the term anchorage refers to methods for preventing undesirable tooth movement. Currently, orthodontic anchorage relies on auxiliary mechanical devices. In some cases, more control is desired than is clinically achieved (Clemmer and Hayes, 1979; Chen et al., 2007; Al-Awadhi et al., 2015; Kim et al., 2016a). As a result, treatment times can be extended and/or an ideal occlusion may not be achieved. Longer treatment times increase the risk for negative sequelae such as decalcification/demineralization, caries and tooth root resorption (Sameshima and Sinclair, 2001; Gonzales et al., 2008; Feldens et al., 2015; Kim et al., 2016b; Pastro et al., 2018).

Orthodontic tooth movement is a bone modeling process in which orthodontic forces shift teeth within sockets causing hypoxia, fluid flow and tissue stretch/compression. These mechanical changes stimulate sterile inflammatory signaling, recruitment and activity of osteoclasts and osteoblasts, and tooth movement beyond the constraints of the original tooth socket (Masella and Meister, 2006; Jiang et al., 2016). Nitric oxide (NO) is a signaling molecule that was previously hypothesized to mediate orthodontic tooth movement (Yoo et al., 2004). NO was originally identified as “endothelial-derived relaxing factor”. NO release from endothelial cells leads to the production of cGMP which has paracrine effects on nearby smooth muscle cells leading to a vasodilation response (Griffith et al., 1984; Ignarro, 1990; Lowenstein and Snyder, 1992). In addition to its vascularization effect (Griffith et al., 1984; Shih and Claffey, 1998), NO has inflammatory and immunomodulatory (Marcinkiewicz and Chain, 1993; Eun et al., 2000) functions. NO is known to alter inflammatory cytokine and chemokine production, alter osteoblast and osteoclast bone anabolism/catabolism, and enhance vascularization and blood flow (Kalyanaraman et al., 2017; Vanhoutte et al., 2017; Gozdzik et al., 2018; Gantner et al., 2020). Physiologic effects of NO are limited by the location and timing of NO production, because NO has a short half-life and small diffusional distance (Lancaster, 1997; Vaughn et al., 1998). Therefore, an NO producing biomaterial is essential for therapeutic application of NO. NO producing biomaterials are currently under investigation for viral infection (Garren et al., 2021), cancer (Khan et al., 2020), inflammation, obesity, cardiovascular disease (Bladowski et al., 2020; Król and Kepinska, 2020), and cervical ripening for birth (Ghosh et al., 2016). Based upon these prior findings, in this study we investigate the efficacy of NO released from S-nitrosothiol containing silica nanoparticles for control of orthodontic tooth movement.

## MATERIALS AND METHODS

### Formulation of Nitric Oxide Releasing Nanoparticles

NO releasing, silica nanoparticles were fabricated at room temperature according to the method previously developed by Frost and Meyerhoff (Frost and Meyerhoff, 2005). Briefly, 7–10 nm fumed silica particles were derivatized with 3-aminopropyltrimethoxsilane to cover the particle surface. The primary amine was reacted with a self-protected thioacetone to form an amide bond and exposes a free thiol. The free thiol was then nitrosated to produce the NO donor, S-Nitroso-N-acetyl-D-penicillamine (SNAP). Residual solvent was removed by air drying of particles under vacuum overnight. Approximately 60 mg of particles were suspended in 1 ml of toluene in a 5 ml glass vial. 300 ml of t-butylnitrite was added. The reaction mixture was agitated, covered in foil and allowed to react for 2 h. The solvent was then removed and particles were vacuum dried. S-nitrosothiol containing silica nanoparticles are highly stable and were stored at room temperature in the dark before use.

### *In Vitro* Release Kinetic Studies

**FIGURE 2 |:**
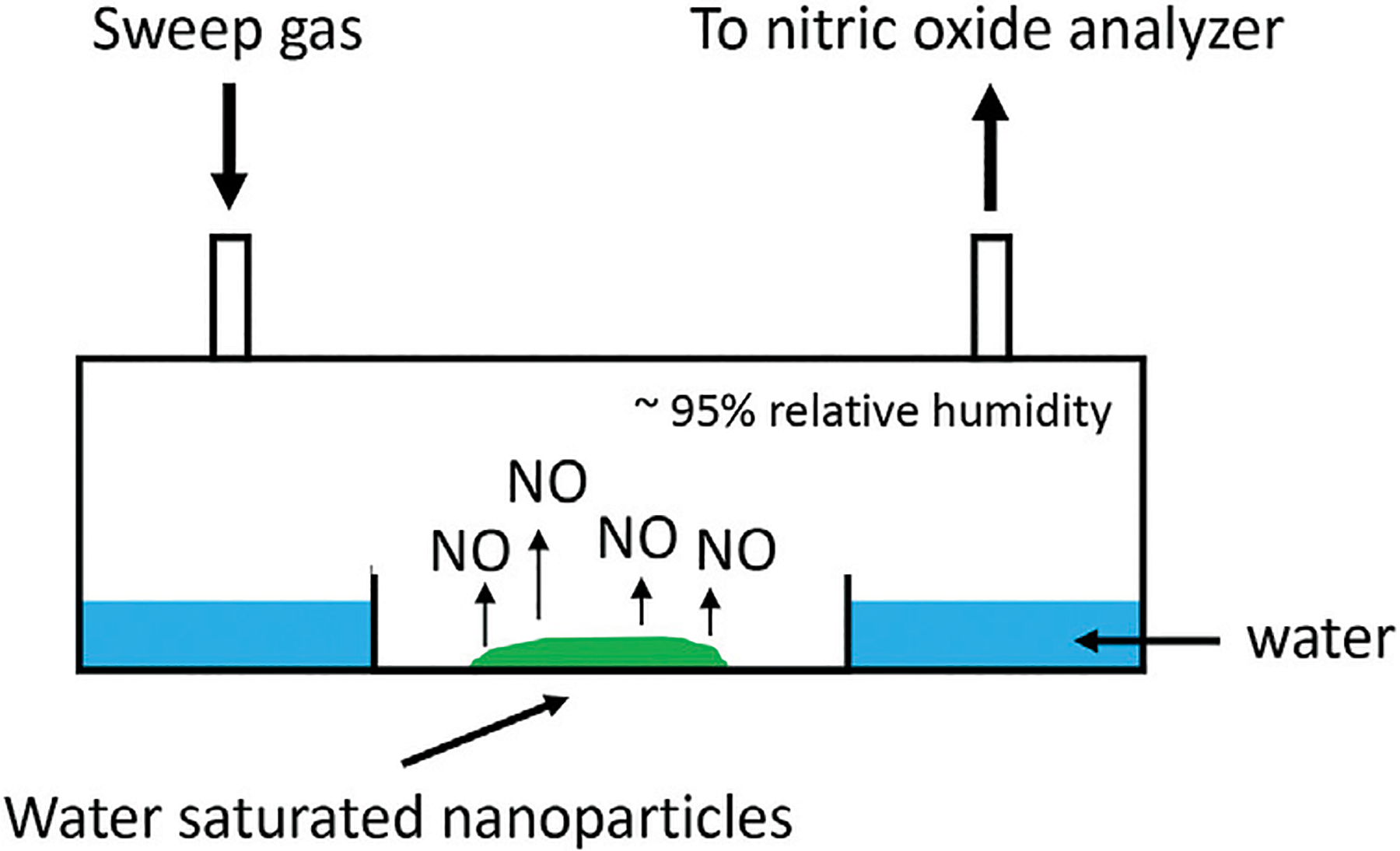
Schematic of *in vitro* NO release kinetic analysis of nanoparticles. Nanoparticles saturated with deionized water released NO into a humidified chamber. NO was measured by chemiluminescence. Sweep gas containing no NO was ambient air.

Release of NO from fabricated nanoparticles was performed on nanoparticles that were saturated with deionized water and held at 37°C in a humidified chamber for the duration of the experiment (Figure 2). NO was quantified by ozone based chemiluminescent detection with Sievers 280i Nitric Oxide Analyzer (NOA) using 200 ml/min flow rate. 45 ppm medical grade calibration NO gas (Air Liquid Healthcare America Corp.) was used to complete the 2-point calibration of the chemiluminescent detector prior to analyses. Ambient air was used to calibrate for zero NO gas and as the sweep gas. NO was swept from the headspace of 60 mm disposable petri dish sample holders. NO measurements were taken every day for 10 min until NO was no longer detected. Data was recorded at an interval of 1/sec.

### Animals and Orthodontic Appliance

In this study, we compared two experimental groups (animals that received an injection with NO releasing nanoparticle but no orthodontic appliances and animals that received an injection with NO releasing nanoparticle plus orthodontic appliances) with two control groups (animals that received an injection with saline but no orthodontic appliances and animals that received an injection with saline plus orthodontic appliances). Rats were purchased from a commercial supplier and acclimatized for 1 week prior to experimentation. Each group was comprised of eight male Sprague-Dawley rats of approximately 350 g who were randomly distributed to each group. This sample size was based upon the variability found in our prior studies in which *n* = 6–8 per group was adequate to establish significant differences in tooth movement between groups (Hudson et al., 2012; Schneider et al., 2015; Sydorak et al., 2019). Rats were housed in a controlled atmosphere of 25°C with a 12-hour light and dark cycle. They were fed a diet consisting of standard rat chow (Harlan Laboratories, Indianapolis, IN) and distilled water *ad libitum*. Based upon past studies, an a priori exclusion criteria was excessive vertical movement of the first molar. No animals had molars with significant extrusion during or after the tooth movement period, likely due to the diligence and regular attention given to the rats and their orthodontic appliances. Animals were anesthetized with isoflurane for the injection and orthodontic appliance placement (and appliance adjustment as needed). Animals were euthanized by CO_2_ overdose.

Just prior to orthodontic appliance placement, each rat was injected with 30 μL of saline (control animals) or saline containing 2.2 mg/kg of nanoparticles containing S-nitrosothiol groups (experimental animals) into the gingival mucosa just mesial to the maxillary first molar teeth immediately prior to orthodontic appliance activation using a 50 ul microliter syringe and 33 gauge needles (Hamilton Company, Reno NV). As previously described (King et al., 1991; Dunn et al., 2007; Sydorak et al., 2019) for the orthodontic appliance, closed coil nickel-titanium springs to provide 50 g mesial molar force were delivered to the maxillary first molars by ligation of between the maxillary first molar and ipsilateral maxillary central incisor with stainless ligatures that were then adhered with composite. Mandibular incisors were filed down on a weekly basis to reduce appliance breakage. Orthodontic appliances were repaired and/or adjusted as needed to accommodate for continuously erupting maxillary incisors so as to maintain a horizontal force vector. All animal procedures followed federal guidelines and were approved of by the University of Michigan Institutional Animal Use and Care Committee prior to the study.

### Measurements of Tooth Movement

Tooth movement was measured every 6 days by stone models made from polyvinylsiloxane impressions, as previously described (Hudson et al., 2012). The occlusal surfaces of each model were scanned (Epson Expression 10,000 XL) at 1200 dpi adjacent to a 10 mm ruler and then magnified at 20 × using imaging software (Adobe Photoshop CS5, Adobe Systems, Inc., San Jose, CA). Measurements were calibrated to the imaged ruler. Tooth movement was measured to the nearest 0.01 mm. Molar mesial movement was measured from the distal groove of the maxillary first molar to the distal surface of the maxillary third molar. Incisor distal movement was measured from the distal surface of the maxillary third molars to the mid-facial gingival margin of the ipsilateral incisor. To account for growth of the maxilla during the 18 days of orthodontic treatment, incisor distal movements were normalized using the incisor position compared to third molar position in animals without orthodontic appliances (average of 1.2 mm). Molar tooth movement did not need normalization as there was no growth displacement of the first relative to the third molar in the animals without orthodontic appliances.

### Micro Computed Tomographic Bone Analyses

**FIGURE 3 |:**
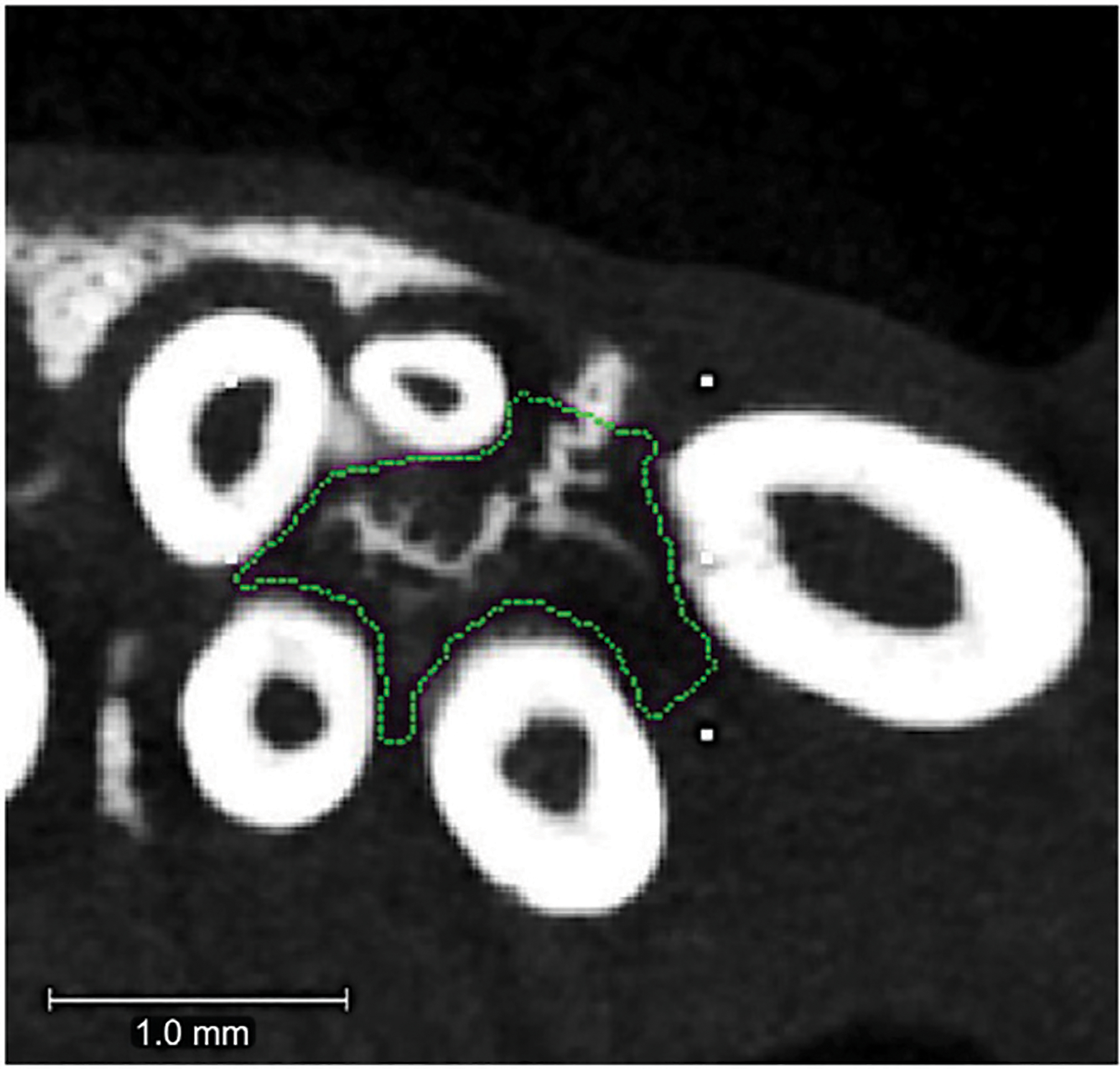
Alveolar bone region of interest. Micro-CT axial slice image showing region of interest for the maxillary first molar intra-radicular alveolar bone. The region of interest (ROI) is outlined in green. This ROI extended from the molar root furcation to the apex of the root and did not include the tooth roots themselves. Tiny white rectangles are orientation marks placed by the operator prior to custom outlining the 3D intra-radicular ROI for analysis.

After 18 days of tooth movement, animals were euthanized by CO_2_ overdose. Micro-CT analyses were performed to quantify alveolar and long bone volume and density between groups. Alveolar bone analysis provides information on potential effects of tooth movement and/or nanoparticles on local bone. Long bone analysis provides information on potential effects of tooth movement and/or nanoparticles on distant bone (systemic effect). Hemi-maxillae and femurs were dissected, fixed in 10% formalin for 48 h followed by transition to 70% ethanol for storage at 4C prior to analyses. Both hemi-maxillae and femur specimens were scanned using a micro-CT system (μCT100 Scanco Medical, Bassersdorf, Switzerland). Scan settings were 18 μm voxel, 70 kVp, 114 μA, 0.5 mm AL filter, and integration time of 500 ms. For femur scans, specimens were scanned over the entire length of the femur. A 0.9 mm section of trabecular bone was analyzed at the proximal metaphysis of the femur, starting 0.9 mm from the growth plate. A 0.9 mm section of cortical bone was analyzed at the mid-diaphyseal shaft. For alveolar bone, an ROI was designated as within the confines of the maxillary first molar roots, from the molar root furcation to the apex of the buccal root (Figure 3). This allowed measurement of bone changes within the local area of the first molar tooth ± injected particles.

### Statistical Analyses

Primary outcomes were quantitative measurements of tooth movements. Secondary outcomes were *in vitro* NO release from fabricated nanoparticles, quantitative measurements of alveolar and femur bone and histomorphometric measurements of root resorption and blood vessels. The sample size was based upon data variability found in our prior studies using this same rodent model of tooth movement (Hudson et al., 2012; Sydorak et al., 2019). Descriptive statistics (mean, standard deviation, 95% confidence intervals) were calculated. Data were checked for normality using the D’Agostino & Pearson test. Normal data were compared using an unpaired t test. Nonnormal data were compared using a Mann-Whitney test. Statistical significance was established as *p* < 0.05.

### Serum Cyclic Guanosine Monophosphate Measurements

Because NO stimulates production of the secondary messenger cyclic guanosine monophosphate (cGMP) (Francis et al., 2010), cGMP can be used as a marker of NO activity (Kalyanaraman et al., 2017). Blood was obtained from animals on day 0 (before orthodontic appliance placement), and days 1, 6 and 12 after orthodontic appliance placement using a 27 gauge needle through the lateral tail vein. Serum was isolated by incubation of blood at room temperature for 40 min followed by centrifugation at 5000 rpm at 4°C. Supernatant (serum) was stored at −80°C until assay. Serum levels of cGMP were measured using a commercially available colorimetric ELISA kit (Abcam, ab133052). This ELISA is composed of a goat anti-rabbit IgG antibody precoated wells, an alkaline phosphatase conjugated-cGMP antigen, and a polyclonal rabbit antibody specific to cGMP. Colorimetric detection of cGMP was measured at 405 nm with a 570 nm correction using a SpectraMax i3 × (Molecular Devices). A standard curve was established using the commercially provided 5,000 pmol/ml stock, which was serially diluted in the commercially provided assay buffer. Final concentrations of 500, 100, 20, 4, 0.8 and 0 pmol/ml were used for standard curve preparation. cGMP levels are reported as pmol/ml by comparison to the standard curve. Due to well number limitations, serum isolated on days 0 and 1 were analyzed separately from serum isolated on days 6 and 12,, resulting in slightly different background levels for the negative control and the experimental samples for days 0 and 1 vs. days 6 and 12. Serum from each animal (*n* = 8 per group) was individually analyzed.

### Quantitative Histomorphometry

**FIGURE 4 |:**
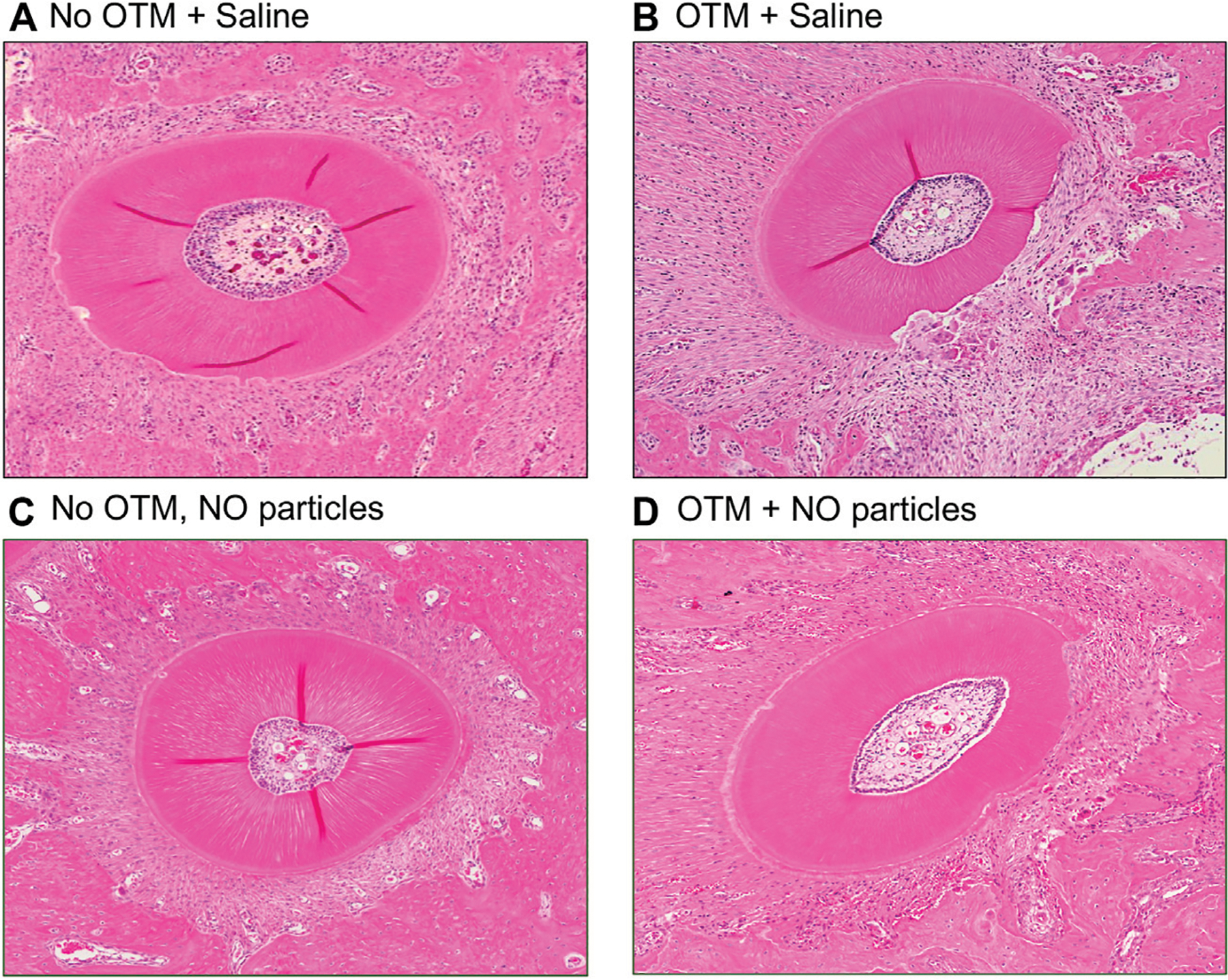
NO releasing biomaterial inhibits molar but not incisor tooth movement. **(A)** Mesial molar tooth movement over the 18-day experimental period. Means ± standard deviations are shown. Animals that had orthodontic appliances plus NO releasing nanoparticles showed significantly diminished mesial molar tooth movement at all time points as compared to animals that had orthodontic appliances but not NO releasing nanoparticles. *p < 0.05, **p < 0.005 between groups. Note diminished slope of tooth movement for animals with NO releasing nanoparticles compared to that for animals without NO releasing nanoparticles from days 1–6 that increases to match that of animals without NO releasing nanoparticles by days 12–18. **(B)** Distal incisor tooth movement at the end of the 18-day experimental period. No significant differences were found for incisor movement between groups (PBS mean = 1.92, NO mean = 1.90, 95% confidence interval of difference between the means = −0.26 to +0.22). Whisker plots showing medians, interquartile range, minimum and maximum values are shown.

Hemi-maxillae were dissected, fixed in 10% neutral buffered formalin and decalcified in 10% EDTA for 6 weeks. 6 mm sections axial sections were taken from the coronal third of the root (Figure 4). We quantified the coronal third of the root to ensure that sections were of similar apical depths by using the molar root furcation as a reference and to view all five roots of the tooth. Sections were stained with hematoxylin and eosin (H&E) for visualization of all roots plus periodontal ligament and surrounding alveolar bone of the first molar tooth. Histomorphometry was performed on the smaller distobuccal and buccal (intermediate) tooth roots, as smaller roots are more prone to resorption due to orthodontic tooth movement (Gonzales et al., 2008; Cuoghi et al., 2014). *ImageJ* software (National Institutes of Health) was utilized to quantify percent resorption per tooth root and number blood vessels within the periodontal ligament (PDL). Roots that were conjoined or otherwise undefined were excluded. This resulted in final buccal root samples sizes of *n* = 8 for control animals with no appliance and saline injections, *n* = 5 for control animals with the orthodontic appliance and saline injections, *n* = 8 for experimental animals with no appliance and nanoparticles injection, and *n* = 6 for experimental animals with the orthodontic appliance and nanoparticles injection. This resulted in final distobuccal root samples sizes of *n* = 8 for control animals with no appliance and saline injections, *n* = 7 for control animals with the orthodontic appliance and saline injections, *n* = 8 for experimental animals with no appliance and nanoparticles injection, and *n* = 7 for experimental animals with the orthodontic appliance and nanoparticles injection. Percent root resorption was calculated using the difference between the estimated original elliptical root area and the actual root area. Blood vessels located within the PDL space were counted and qualified as compressed or not compressed. Three separate axial sections from each hemi-maxilla were analyzed then averaged per animal for comparison across groups.

## RESULTS

### Animal Status

One animal died during the initial appliance placement procedure due to an adverse reaction to the anesthesia and was excluded from the study. This animal was replaced with another rat. All other animals were included with no adverse reactions to anesthesia, appliance placement or tooth movement, and followed until day 18 when they were euthanized, such that final animal numbers for tooth movement and micro-CT measurements were *n* = 8 per group. No significant differences in initial animal weights were seen between any of the groups. Prior to injections and orthodontic appliance placement, animals with orthodontic appliances weighed 359 ± 20 g (mean ± standard deviation), and 379 ± 13 g in the PBS and nanoparticle groups, respectively. Prior to injections, animals without orthodontic appliances weighed 364 ± 15 g (mean ± standard deviation), and 383 ± 27 g in the PBS and nanoparticle groups, respectively. At day 18, animals with orthodontic appliances weighed 427 ± 18 g, and 427 ± 29 g in the PBS and nanoparticle groups, respectively. At day 18, the animals without orthodontic appliances weighed 441 ± 31 g and 467.6 ± 30 g in the PBS and NO nanoparticle groups, respectively. No significant differences in final animal weights were seen between any of the groups.

### Tooth Movement

**FIGURE 5 |:**
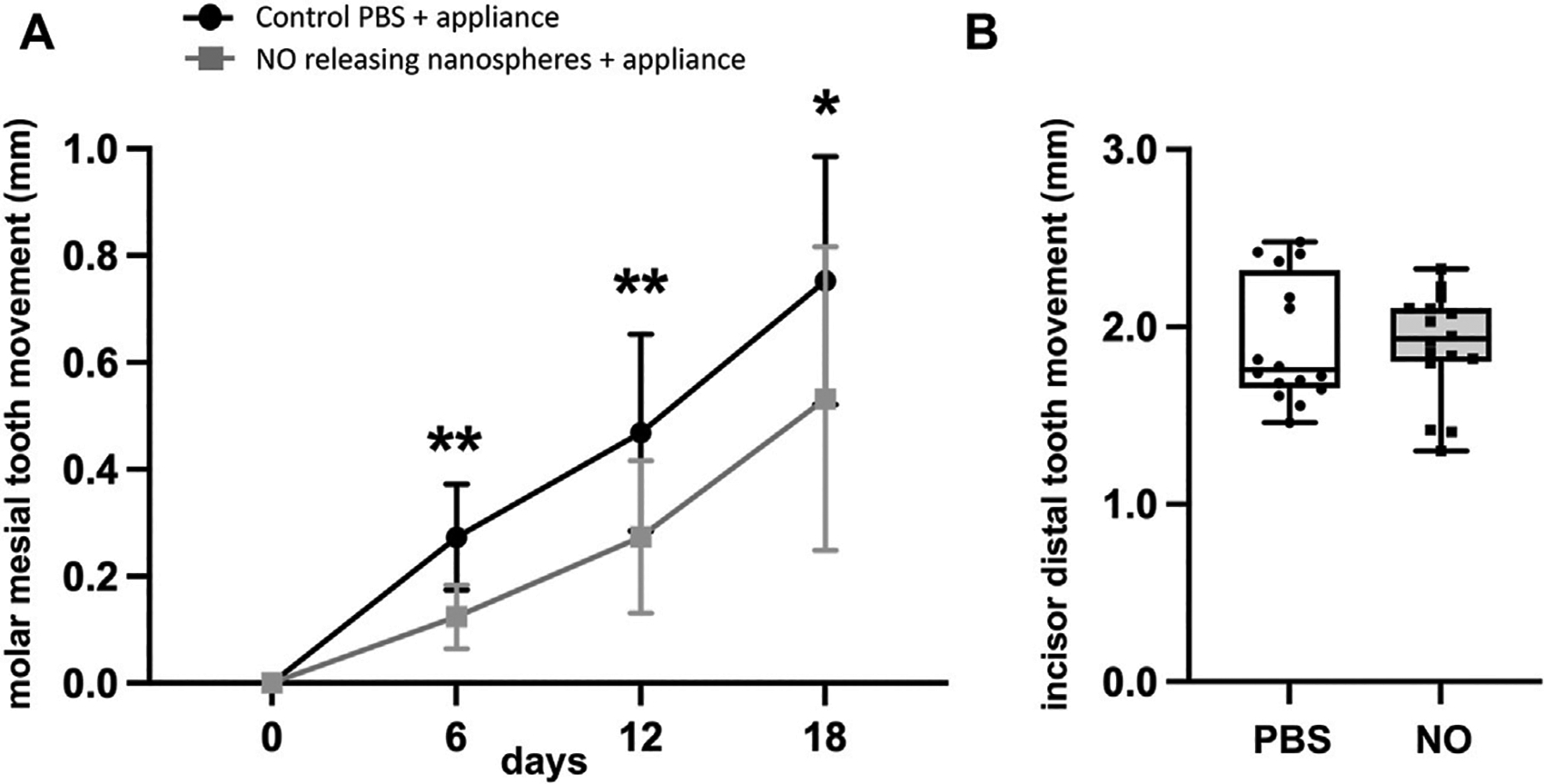
Histology of the distobuccal root after 18 days ± orthodontic tooth movement. Hematoxylin and eosin (H&E) stained representative images of the distobuccal root of the first molar of **(A)** animals that received control saline injections and no orthodontic appliances, **(B)** animals that received control saline injections and had orthodontic appliances, **(C)** animals that received experimental nanoparticles injections and no orthodontic appliances, and **(D)** animals that received experimental nanoparticles injections and had orthodontic appliances. OTM = orthodontic tooth movement, saline = PBS = phosphate buffered saline.

As stated above, experimental animals that received an injection of the NO releasing nanoparticles included a group that had orthodontic appliances and a group that did not have orthodontic appliances. Control animals that received a single injection of saline also included a group that had orthodontic appliances and a group that did not have orthodontic appliances. All injections were performed into the gingival mucosa just mesial to the maxillary first molar tooth just prior to orthodontic appliance placement. The single injection of 2.2 mg/kg nanoparticles significantly reduced the amount of mesial molar tooth movement as compared to the amount of cumulative mesial molar tooth movement seen in animals that received saline injections at day 6 (0.12 ± 0.01 vs. 0.27 ± 0.02 mm, *p* < 0.001), day 12 (0.27 ± 0.04 vs. 0.47 ± 0.05 mm, *p* < 0.01) and day 18 (0.53 ± 0.07 vs. 0.75 ± 0.06 mm, *p* < 0.05) (Figure 5A). The single injection of NO releasing silica nanoparticles did not significantly influence the amount of distal incisor tooth movement as compared to that seen in animals that received saline injections (day 18, 1.90 ± 0.08 vs. 1.92 ± 0.08, not significant) (Figure 5B).

### Silica Nanoparticle Nitric Oxide Release Kinetics and Relationship to Tooth Movement

**FIGURE 6 |:**
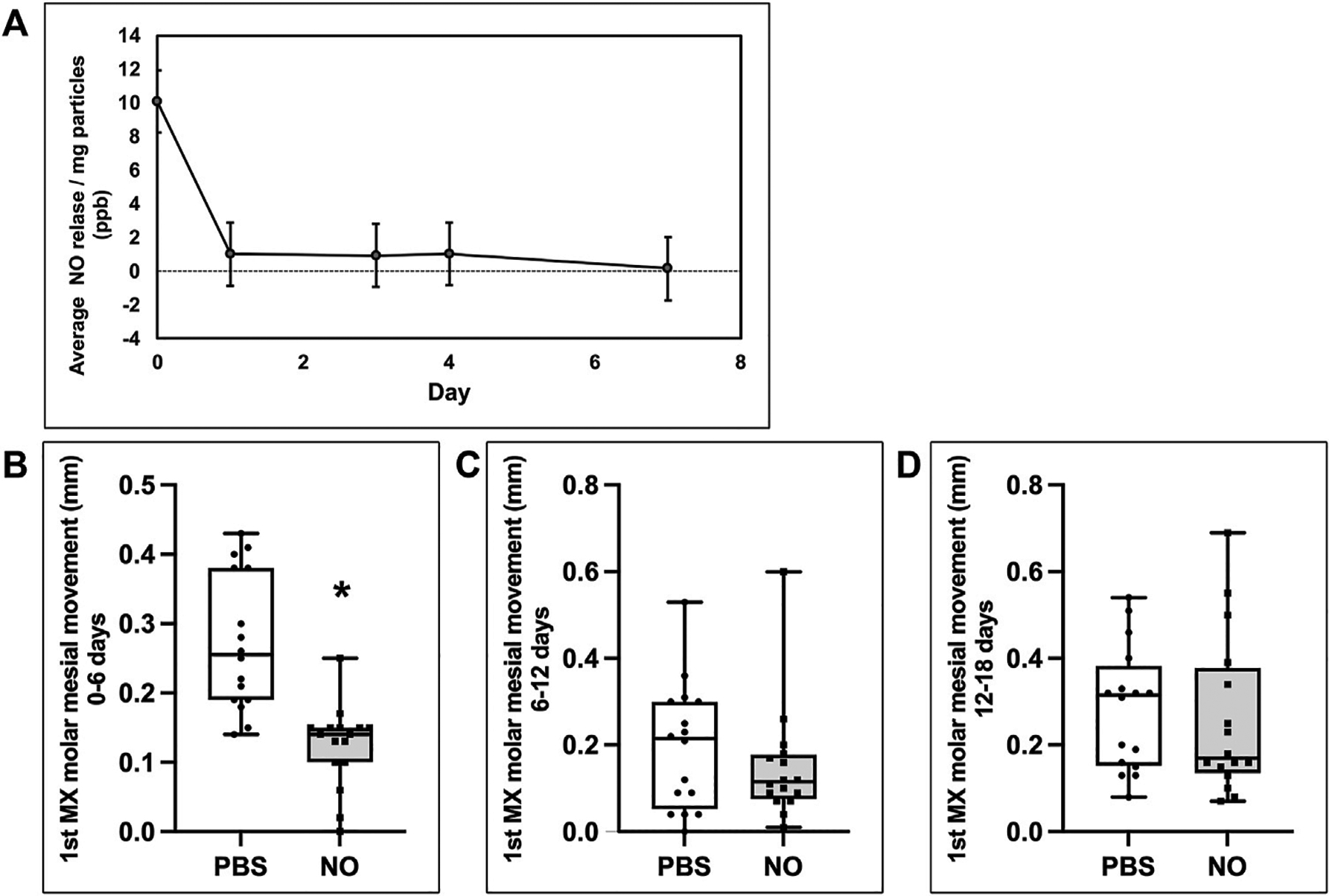
Inhibition of molar tooth movement coincides with NO release from N-nitrosothiol nanoparticles. **(A)** NO *in vitro* release profile from nanoparticles. Results show an initial burst release followed by stable NO release until day 4 upon which NO release decreases to non-measurable levels by day 7. **(B,C,D)** Mesial molar tooth movements on days 1–6 **(B)**, 6–12 **(C)** and 12–18 **(D)**. Whisker plots showing medians, interquartile range, minimum and maximum values are shown. Mesial molar tooth movement is significantly inhibited by over 50% during days 1–6 of the experimental period. *p < 0.001. No significant inhibition of mesial molar tooth movement is seen during days 6–12 or days 12–18 of the experimental period.

*In vitro* release kinetic studies using the same batch of formulated nanoparticles that was used *in vivo* revealed that the nanoparticles released a burst of NO within the first hours at 10.0 ± 0.32 ppb/mg particles (1.8^−12^ ± 5.8^−14^ nmol/mg particles that diminished by 10x to 1.0 ± 0.05 ppb/mg particles (1.8^−13^ ± 8.6^−15^ nmol/mg particles over the next 1–4 days, and then diminished again by tenfold from day 4 to day 7, at which point it was no longer measurable (Figure 6A).

Based upon the *in vitro* release data showing loss of NO release by approximately day 7 *in vitro*, it seemed likely that the effect of the NO releasing nanoparticles was likely lost by the final time point of 18 days *in vivo*. Therefore, to gain a better appreciation for orthodontic anchorage (inhibition of mesial molar movement without any effect on distal incisor movement) at earlier time points, we calculated the amount of molar movement separately, between days 0–6, 6–12 and 12–18. While cumulative tooth movement was significant at all time points, comparisons of intermediate time periods (0–6, 6–12, and 12–18) show that tooth inhibition in the NO releasing group occurred only during days 0–6, with no significant inhibition noted between days 6–12 or 12–18 (Figures 6B–D). This is also evident when viewing the cumulative molar tooth movement data in that the difference between slopes of the experimental (received NO nanoparticles) and control (received saline) groups diminished over time (Figure 5A). That tooth movement returned to control rates after release of NO is worth noting because this indicates that no rebound effect occurred after cessation of exposure to NO. From this data, we also calculated the average percent inhibition of mesial molar tooth movement in the NO releasing nanoparticle group compared with the control group. Animals injected with the nanoparticles had 54% less molar movement at day 6, 42% less cumulative molar movement at day 12 and 29% less cumulative molar movement at day 18.

### Local Alveolar Bone Parameters

To evaluate local effects of the delivered NO releasing biomaterial on alveolar bone investing the tooth adjacent to the site of injection and under orthodontic forces, first molar intra-radicular bone quality parameters were measured via micro CT. Sample size was *n* = 8 specimen per group. No differences in bone volume fraction, bone mineral density or tissue mineral density were seen between animals that received saline vs. animals that received that NO releasing nanoparticles, regardless of orthodontic appliance placement, at day 18 of the tooth movement experiments (Table 1). As expected, bone volume fraction, bone mineral density and tissue mineral density were significantly reduced in animals with orthodontic appliances compared to those without orthodontic appliances, regardless of NO delivery.

### Long Bone Parameters

To evaluate potential systemic effects that local injections of the experimental NO releasing biomaterial might have on bones distant from the site of injection, femur cortical and trabecular bone were analyzed by micro CT. Sample size was *n* = 8 specimen per group. No differences in trabecular bone volume fraction, trabecular number, trabecular thickness or trabecular spacing were seen between animals that received saline vs. animals that received that NO releasing nanoparticles, regardless of orthodontic appliance placement, at day 18 of the tooth movement experiments (Table 2). One parameter (trabecular bone volume fraction) was significantly diminished in animals with appliances compared to animals without appliances in the NO releasing nanoparticle groups. No differences in cortical bone volume fraction, bone mineral density and tissue mineral density were seen between animals that received saline vs. animals that received that NO releasing nanoparticles. In addition, no differences in cortical bone parameters were seen between animals with orthodontics appliances and animals without orthodontic appliances (Table 3).

### Serum Cyclic Guanosine Monophosphate Measurements

Because NO cannot be directly measured *in vivo*, to determine if the injected NO releasing nanoparticles altered NO signaling systemically, we measured serum levels of cGMP, the primary direct downstream target of NO (Friebe and Koesling, 2009). Results show that no change in circulating cGMP levels occurred in any of the control or experimental groups, before or after injection of the NO releasing nanoparticles (Supplementary Figure S1).

### Root Resorption and Vascularization

**FIGURE 7 |:**
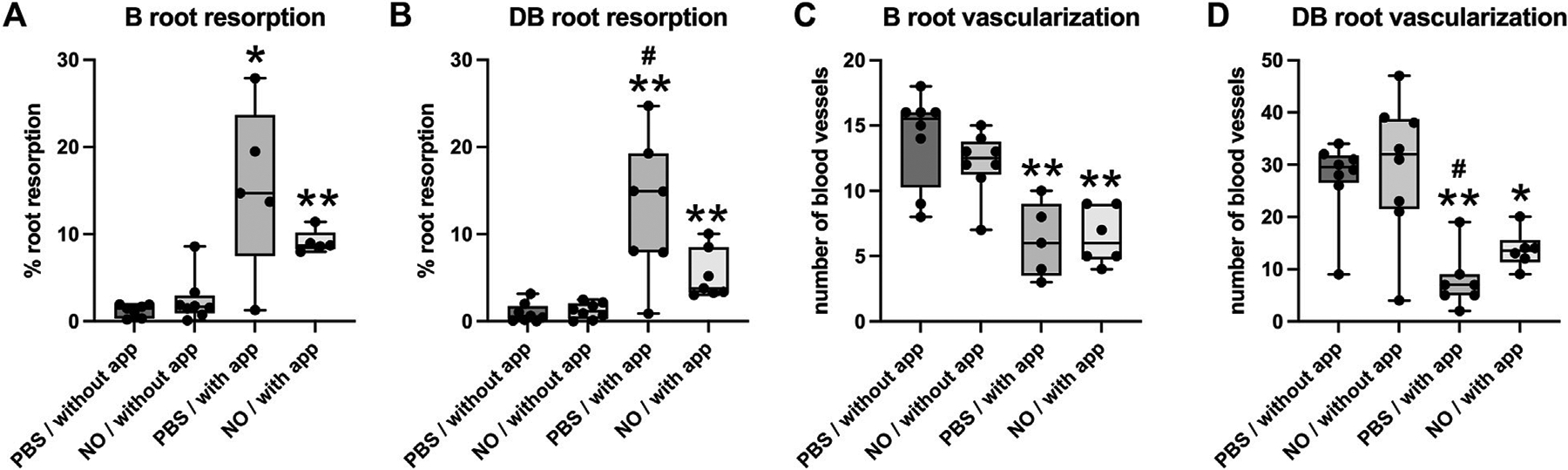
Histomorphometry of root resorption and PDL vascularization of the buccal and distobuccal roots of the maxillary first molar after 18 days ± orthodontic tooth movement. Whisker plots showing medians, interquartile range, minimum and maximum values are shown. **(A,B)** Root resorption is significantly increased in animals that underwent tooth movement compared to those that did not have orthodontic appliances on both buccal and distobuccal roots. **(C,D)** Blood vessel numbers in the PDL were significantly decreased in animals that underwent tooth movement compared to those that did not have orthodontic appliances on both buccal and distobuccal roots. Significantly less root resorption was seen in animals that underwent tooth movement and received injections of NO releasing nanoparticles compared to animals that underwent tooth movement and received injections of saline for the distobuccal root **(B)**. Significantly more blood vessels were seen in animals that underwent tooth movement and received injections of NO releasing nanoparticles compared to animals that underwent tooth movement and received injections of saline for the distobuccal root **(D)**. *p < 0.05 compared to group without tooth movement, **p < 0.005 compared to group without tooth movement, ^#^*p* < 0.05 compared to group that received nanoparticle injections. Without app = no orthodontic appliances/no orthodontic tooth movement, with app = with orthodontic appliances/with orthodontic tooth movement, PBS = phosphate buffered saline.

Histomorphometric measurements of root resorption revealed significantly greater resorption of distobuccal and buccal (intermediate) tooth roots in animals with orthodontic appliances as compared to animals without orthodontic appliance (Figure 7). In addition, resorption of the distobuccal root was significantly lower in animals with appliances that were injected with the NO releasing nanoparticles as compared to animals with appliances that did not receive injections with the nanoparticles.

Blood vessel quantification revealed that the number of blood vessels located within the PDL of distobuccal and buccal (intermediate) tooth roots was significantly lower in animals with orthodontic appliances as compared to animals without orthodontic appliance. In addition, the number of PDL blood vessels within the distobuccal root was significantly higher in animals with appliances that were injected with the NO releasing nanoparticles as compared to animals with appliances that did not receive injections with the nanoparticles (Figure 7). Blood vessels were compressed in all animals with orthodontic appliances and were not compressed in animals with no appliances.

## DISCUSSION

Because no fixed intraoral anatomical anchor exists, orthodontic forces can create both desirable and undesirable tooth movements. During orthodontic treatment, precisely controlled anchorage (inhibition of undesired tooth movement to enhance favorable tooth movement) is often critical to effectively establish a stable, highly functional occlusion. Contemporary orthodontists utilize various mechanical techniques to enhance orthodontic anchorage, but such techniques can lead to negative side effects, and are dependent upon patient compliance and/or an appropriate anatomic location for placement (Cole, 2002; Jambi et al., 2013; Janson et al., 2013; Jambi et al., 2014; Kuroda and Tanaka, 2014; Al-Awadhi et al., 2015; Arponen et al., 2020). In addition, even appliances that utilize skeletal anchorage, such as temporary anchorage devices (TADs), while effective, do not provide 100% anchorage (Jambi et al., 2014; Kaipatur et al., 2014; Becker et al., 2018). Biological methods which influence orthodontic tooth movement at a cell and molecular level could provide alternative adjunctive methods for inhibition of undesired tooth movement.

Nitric Oxide (NO) plays a critical role in orthodontic tooth movement. Expression of nitric oxide synthase (NOS) isoforms iNOS and eNOS increase in PDL cells and local osteocytes upon application of orthodontic force (Nilforoushan and Manolson, 2009; Tan et al., 2009). This is not surprising given that tooth movement leads to fluid shear stress on local PDL cells and osteocytes, and shear stress stimulates expression of eNOS and NO production (Fox et al., 1996; Zaman et al., 1999; Davis et al., 2001; Kalyanaraman et al., 2018). Orthodontic tooth movement also stimulates the expression of inflammatory cytokines (Lowney et al., 1995; Uematsu et al., 1996; Alhashimi et al., 2001; Kaku et al., 2008), and cytokines stimulate expression of iNOS and NO production (Ralston et al., 1995; Huang, 2000). Previous studies showed that local injection of L-arginine (NO precursor) increased tooth movement in rats (Shirazi et al., 2002; Akin et al., 2004), while local injection of L-NAME (NOS inhibitor) inhibited tooth movement in rats (Hayashi et al., 2002; Shirazi et al., 2002). These NO manipulation during tooth movement studies appear to indicate that stimulation of NO production is bone catabolic, enhancing osteoclast activity and orthodontic tooth movement. Yet, it is well known that the efficacy of manipulating NO through these methods (delivery of L-arginine and/or L-NAME) is likely inadequate for optimal changes in NO efficacy (Anastasio et al., 2020). For this reason, here we utilized an NO producing biomaterial to establish NO effects on tooth movement.

In this study we show for the first time that nitric oxide (NO) delivery via injected NO releasing nanoparticles inhibits orthodontic movement of teeth adjacent to the site of injection. This tooth movement effect is likely due to the bone anabolic effects of NO. Our results are consistent with other reports showing that endogenous and exogenous NO has bone anabolic effects. Genetic ablation of eNOS in mice leads to decreased bone mineral density and cortical thinning with reduced osteoblast numbers and mineral apposition rate (Armour et al., 2001). Genetic ablation of iNOS in mice with hind limb suspension (mechanical unloading) do not increase bone formation after reloading (Watanuki et al., 2002). Genetic ablation of nNOS leads to diminished trabecular bone mineral density with reduced bone remodeling by 10 weeks in mice (van’t Hof et al., 2004). Together, these studies indicate that loss of any of the three NOS isoforms and therefore NO production, diminishes bone anabolism. Other studies also showed that NO is an essential mediator of the bone anabolic effects of both estrogen and mechanical loading in mice (Armour et al., 2001; Rangaswami et al., 2009; Rangaswami et al., 2010; Marathe et al., 2012). NO releasing biomaterials were more recently proposed for better treatment of bone fractures and osteoporosis (Anastasio et al., 2020). In addition, of particular relevance to the current study, intraperitoneal injections of an NO producing biomaterial into female ovariectomized mice was shown to increase cGMP levels and downstream signaling, increase osteoprogenitor proliferation, increase osteoblast gene expression, reduce osteocyte apoptosis, reduce osteoclast numbers and reduce bone resorption. Overall, the NO biomaterial treated mice had significantly increased trabecular bone mass when compared to non-treated mice (Kalyanaraman et al., 2017), demonstrating that NO delivered via NO releasing biomaterials are bone anabolic, not catabolic, and would therefore be inhibitory to tooth movement.

Orthodontists and patients want efficient and consistently successful treatment outcomes. The results shown here provide a novel approach for control of tooth movement based upon emerging technologies for drug delivery and advances in our knowledge of biologic processes that control orthodontic tooth movement. Results of this study show that injection of the NO releasing nanoparticles inhibited tooth movement by over 50% for approximately 1 week after injection. While these results are striking, this study does have limitations, including the need for earlier time points for bone and tissue analyses. *In vitro* release data of NO indicated loss of NO release from nanoparticles after approximately 1 week, but this data was calculated later, leading the *in vivo* study to last for 18 days of orthodontic tooth movement. This resulted in assay of isolated tissues that had not been exposed to NO for approximately 2 weeks. In addition to the need for assay of tissues during NO release, future studies should also investigate utilization of lower force levels in the rodent model (Ren et al., 2004), utilize nanoparticles that do not release NO as a better control, and include a deeper characterization including electron microscopy of the nanoparticles and the nanoparticles-tissue interaction. It is also important to recognize that the NO *in vitro* release studies performed here were performed in deionized water. NO release from biomaterials is influenced by environmental conditions (He and Frost, 2016) such that NO release levels *in vivo* are likely different from that seen *in vitro*. In future studies it will also be important to perform cytotoxicity studies, as nano size silica nanoparticles may exhibit toxicity (Murugadoss et al., 2017). In future studies we plan to utilize biocompatible and biodegradable polymer nanoparticles for delivery of NO producing S-nitrosothiol groups (Kapoor et al., 2015; Elmowafy et al., 2019) to avoid silica based toxicity. Regardless, toxicology studies should be performed regardless of the nanoparticle material used.

The nanoparticles used in this study were configured to incorporate the NO generating S-nitrosothiol molecules but not configured for control of burst release, amount of NO release or duration of release. Results shown here demonstrate that NO was released from the nanoparticles at an initial burst level of 10 ppb/mg particles that decreased by 10x to steady state levels of 1 ppb/mg particles over the next 1–4 days, and then diminished again by tenfold from day 4 to day 7, at which point it was no longer measurable. The burst release was likely generated by trace transition metals that are ubiquitous in physiological environments. In future studies we aim to eliminate the initial burst release by formulating the nanoparticles with aqueous solution containing the chelating agent EDTA (ethylenediaminetetraacetic acid) and/or by coating with a thin film of PLGA (polylactic-co-glycolic acid). We also aim to increase nanoparticle loading of S-nitrosothiol particles to increase NO release levels and to incorporate controlled triggers for NO release, to increase efficacy and control longevity of effect. Of great importance to potential clinical translation, results also showed that inhibition of molar tooth movement only occurred during the period of NO release. After NO release, tooth movement returned to rates seen in control animals. No rebound effect (Teixeira, 2013; Anastasilakis et al., 2021) (increased rate of tooth movement) after end of NO release was seen.

As stated previously, NO influences the production of inflammatory signaling mediators, alters bone cell function, and enhances vascularization. All of these processes are potentially relevant to orthodontic tooth movement. The histomorphometry results shown here indicate that NO release from the nanoparticles significantly increased the number of blood vessels in the PDL of the distobuccal root of the maxillary first molar during tooth movement. While such results were not found for the buccal root, this was likely due to the fact that a smaller sample size was available for buccal root blood vessel quantification due to challenges in definition of the PDL space around this tooth root in animals that had orthodontic appliances. The increased blood vessel count around the distobuccal root in animals with the NO releasing nanoparticles indicates that the NO releasing nanoparticles had vascular effects. NO induces vasodilation, vasopermeability and angiogenesis (Rajendran et al., 2019). The results shown here indicate that the inhibitory tooth movement effects of the NO releasing nanoparticles are likely due to increased blood flow leading to increased oxygen tension in local tissues. Such an effect would decrease the local hypoxia that is induced upon orthodontic tooth movement, leading to diminished induction of downstream signaling and diminished orthodontic tooth movement. Future more comprehensive studies are required to definitively establish that changes in hypoxic signaling mediate the tooth movement properties of the NO releasing nanoparticles.

## Supplementary Material

Serum cGMP levels **Supplementary Figure S1 |** Serum cGMP Levels. cGMP levels in serum at indicated time points were measured by ELISA as measure of NO signaling. Day 0 is prior to ± orthodontic appliance placement. Days 1, 6, and 12 are after ± orthodontic appliance placement. Results show that cGMP levels were not evident in any group at any time point, are always below the negative control data point. Negative and positive control data points differ between days 0, 1 and days 6, 12 because the ELISA had to be performed on two different plates due to well number limitations.

## Figures and Tables

**TABLE 1 |:** Intra-radicular alveolar bone quality measures.

	Bone volume fraction (%)	Bone mineral density (mg/cc)	Tissue mineral density (mg/cc)
No appliances	Mean ± SD: 0.70 ± 0.03 95% CI: 0.68 0.72	Mean ± SD: 714 ± 31 95% CI: 686–743	Mean ± SD: 999 ± 15 95% CI: 985–1021
No Appliances + NO releasing nanoparticles	Mean ± SD: 0.72 ± 0.04 95% CI: 0.67–0.76	Mean ± SD: 736 ± 41 95% CI: 685–787	Mean ± SD: 1004 ± 19 95% CI: 981–1027
Appliances	Mean ± SD: 0.34 ± 0.19** 95% CI: 0.11–0.57	Mean ± SD: 372 ± 170** 95% CI: 162–583	Mean ± SD: 950 ± 29** 95% CI: 913–986
Appliances + NO releasing nanoparticles	Mean ± SD: 0.45 ± 0.08** 95% CI: 0.38–0.52	Mean ± SD: 475 ± 77** 95% CI: 411–539	Mean ± SD: 979 ± 13* 95% CI: 968–990

* p < *.005,*

** p < *.001 vs. no appliances*.

**TABLE 2 |:** Trabecular bone quality measures.

	Bone volume fraction (%)	Trabecular number (1/mm)	Trabecular thickness (mm)	Trabecular separation (mm)
No appliances	Mean ± SD: 0.31 ± 0.07 95% CI: 0.25–0.37	Mean ± SD: 4.7 ± 0.6 95% CI: 4.3–5.2	Mean ± SD: 0.088 ± 0.006 95% CI: 0.83–0.93	Mean ± SD: 0.20 ± 0.03 95% CI: 0.17–0.22
No Appliances + NO releasing nanoparticles	Mean ± SD: 0.34 ± 0.06 95% CI: 0.30–0.39	Mean ± SD: 4.8 ± 0.6 95% CI: 4.3–5.3	Mean ± SD: 0.093 ± 0.004 95% CI: 0.90–0.95	Mean ± SD: 0.19 ± 0.04 95% CI: 0.16–0.22
Appliances	Mean ± SD: 0.35 ± 0.06 95% CI: 0.30–0.40	Mean ± SD: 4.8 ± 0.5 95% CI: 4.4–5.2	Mean ± SD: 0.094 ± 0.007 95% CI: 0.89–0.100	Mean ± SD: 0.19 ± 0.04 95% CI: 0.17–0.21
Appliances + NO releasing nanoparticles	Mean ± SD: 0.30 ± 0.03 95% CI: 0.27–0.32	Mean ± SD: 4.4 ± 0.4 95% CI: 4.1–4.8	Mean ± SD: 0.089 ± 0.003 95% CI: 0.96–0.91	Mean ± SD: 0.21 ± 0.03 95% CI: 0.19–0.24

No significant differences.

**TABLE 3 |:** Cortical bone quality measures.

	Bone volume fraction (%)	Bone mineral density (mg/cc)	Tissue mineral density (mg/cc)
No appliances	Mean ± SD: 0.62 ± 0.03 95% CI: 0.59–0.64	Mean ± SD: 731 ± 26 95% CI: 709–753	Mean ± SD: 1141 ± 17 95% CI: 1126–1155
No Appliances + NO releasing nanoparticles	Mean ± SD: 0.63 ± 0.03 95% CI: 0.60–0.65	Mean ± SD: 746 ± 38 95% CI: 714–778	Mean ± SD: 1146 ± 23 95% CI: 1127–1165
Appliances	Mean ± SD: 0.60 ± 0.03 95% CI: 0.58–0.63	Mean ± SD: 718 ± 33 95% CI: 690–746	Mean ± SD: 1145 ± 20 95% CI: 1129–1161
Appliances + NO releasing nanoparticles	Mean ± SD: 0.60 ± 0.02 95% CI: 0.59–0.62	Mean ± SD: 716 ± 18 95% CI: 701 −731	Mean ± SD: 1139 ± 13 95% CI: 1127–1150

No significant differences.

## Data Availability

The raw data supporting the conclusion of this article will be made available by the authors, without undue reservation.
