# Supplementary material for: Control of Orthodontic Tooth Movement by Nitric Oxide Releasing Nanoparticles in Sprague-Dawley Rats
Source: Front Dent Med. Author manuscript; Available in PMC 2022 Sep 7. (PMC9451041; doi:10.3389/fmats.2022.811251)

Supplemental Figure 1. Serum cGMP Levels. cGMP levels in serum at indicated time points were measured by ELISA as measure of NO signaling. Day 0 is prior to +/- orthodontic appliance placement. Days 1, 6, and 12 are after +/- orthodontic appliance placement. Results show that positive cGMP levels were not evident in any group at any time point and are always below the negative control data point. Negative and positive control data points differ between days 0, 1 and days 6,12 because the ELISA had to be performed on two different plates due to well number limitations.

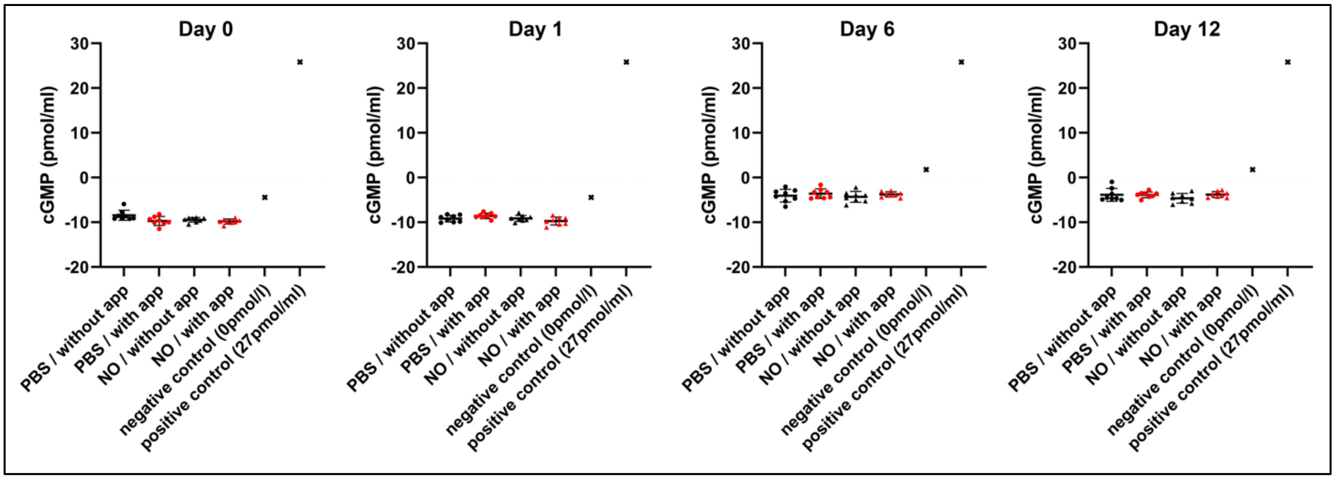

Supplement: Serum cGMP levels — Supplementary Figure S1 | Serum cGMP Levels. cGMP levels in serum at indicated time points were measured by ELISA as measure of NO signaling. Day 0 is prior to ± orthodontic appliance placement. Days 1, 6, and 12 are after ± orthodontic appliance placement. Results show that cGMP levels were not evident in any group at any time point, are always below the negative control data point. Negative and positive control data points differ between days 0, 1 and days 6, 12 because the ELISA had to be performed on two different plates due to well number limitations. [file NIHMS1831360-supplement-Serum_cGMP_levels.pdf]
